# Glioblastoma insights: Focus on crosstalk with the suppressive tumor microenvironment and advanced therapeutic strategies

**DOI:** 10.1097/MD.0000000000046285

**Published:** 2026-05-12

**Authors:** Yi Wei, Yuwei Shi, Hu Wang, Yinwu Shang, Bingwen Yu, Yunjuan Zhang

**Affiliations:** a Neurosurgery Department, Gansu Provincial People’s Hospital, Lanzhou, China; b Gansu University of Chinese Medicine, Lanzhou, China; c Gansu Provincial People’s Hospital, Lanzhou, China.

**Keywords:** chimeric antigen receptor, crosstalk, glioblastoma, immune checkpoint inhibitors, tumor microenvironment

## Abstract

Glioblastoma (GBM) is an extremely aggressive brain tumor for which there is currently no curative therapy available. The tumor microenvironment comprises an intricate interplay of various cells and molecules. Recent research has increasingly emphasized the inhibitory characteristics of the microenvironment, which significantly influence tumor growth, proliferation, and response to therapy through mechanisms including immunosuppression, hypoxia, and nutritional deficits. Comprehension of the crosstalk between GBM and the suppressive tumor microenvironment has the potential to advance future investigations aimed at surmounting this inhibitory microenvironment. Additionally, it could offer guidance for the formulation of novel therapeutic approaches. Significantly, we reviewed advanced therapeutic strategies to overcome GBM, such as immune checkpoint inhibitors, chimeric antigen receptor therapies, nanoparticles, small molecule inhibitors, and stem cell-based therapies. These strategies are continuously evolving and being improved, with the potential to significantly augment the therapeutic effectiveness against GBM.

## 1. Introduction

As a multifaceted systemic illness, the clinical management of cancer has progressed from conventional approaches such as surgical resection, radiotherapy, and chemotherapy to a comprehensive, multimodal treatment framework. In recent years, advancements in biomedical technology have led to the emergence of novel therapeutic approaches, including exosome drug delivery systems, CRISPR-Cas9 gene editing, and immunomodulatory biomaterials, which offer new strategies for cancer intervention.^[1–3]^ Given the heterogeneous and stage-specific nature of cancer progression, the development of individualized combinatorial therapeutic procedures has emerged as a significant focus in clinical practice.

Glioblastoma (GBM) represents the most prevalent primary brain tumor in the adult population.^[4,5]^ In recent times, significant progress has been achieved in understanding the pathology, early detection, and strategic selection of combination therapy for GBM. However, the formidable aggressiveness of GBM results in persistently low survival rates for patients, positioning it as one of the malignancies with the most unfavorable 5-year survival outcomes.^[6–9]^

The prevailing treatment regimen for GBM involves prioritizing extensive surgical resection, complemented by a blend of radiotherapy, systemic therapy (chemotherapy and targeted therapy), and supportive care. Nevertheless, each of these strategies presents distinct challenges. Primarily, the invasive character of GBM often results in incomplete tumor removal during surgical resection.^[8]^ Additionally, potential resistance to chemotherapeutic medications renders GBM inherently resistant to them.^[10]^ The existence of the blood–brain barrier (BBB) poses an additional challenge to treatment, hindering the attainment of therapeutic drug concentrations at the tumor site.^[11]^ Immunotherapy has emerged as a promising strategy for treating cancer, as it involves activating or enhancing the body’s immune system to identify and eliminate cancerous cells.^[12,13]^ In recent years, immune-based therapies, such as immune checkpoint inhibitors (ICIs), cytokine therapies, and tumor vaccines, have demonstrated significant improvements in survival rates for various types of solid tumors, such as melanoma and non-small cell lung cancer. However, these treatments have not shown the same success in patients with recurrent GBM.^[14]^ One important consideration is the existence of a suppressive microenvironment (immune, hypoxic, and metabolic microenvironment) in solid tumors that renders immune cells dysfunctional.^[15,16]^ The inherent heterogeneity and limited ability to provoke an immune response of GBM also present a significant obstacle in its treatment.^[17–19]^ The interactions and crosstalk within the suppressive surroundings in the GBM tumor microenvironment (TME) are being studied to offer valuable perspectives on addressing the formidable and intricate obstacle of GBM.

**Figure 1.**
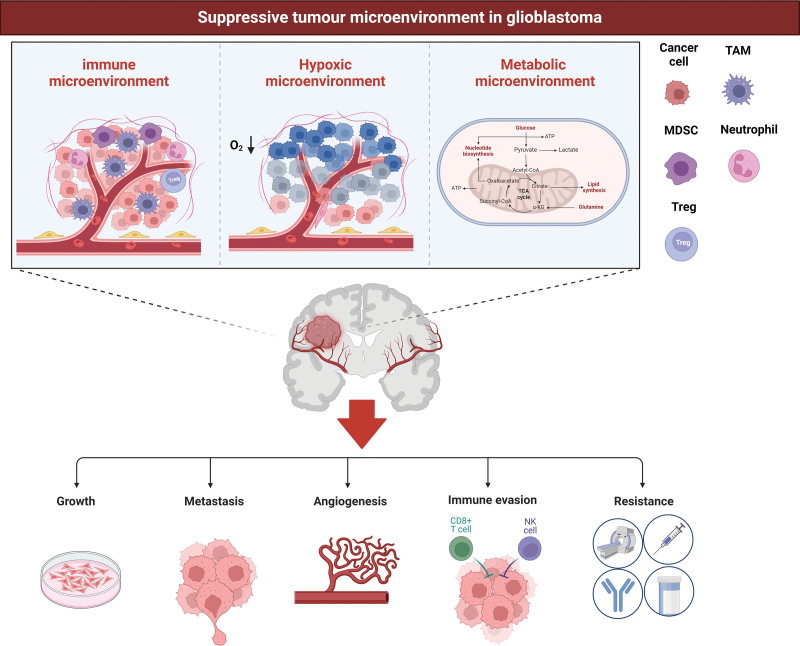
Suppressive tumor microenvironment in glioblastoma. In the microenvironment of glioblastoma (GBM), various cell types, such as tumor cells, tumor-associated macrophages (TAMs), myeloid-derived suppressor cells (MDSCs), neutrophils, and regulatory T cells (Tregs), collaborate to create an immunosuppressive microenvironment. Furthermore, the hypoxic conditions and inadequate nutrition within the GBM contribute to the promotion of tumor growth, metastasis, angiogenesis, immune evasion, and resistance to treatment.

**Figure 2.**
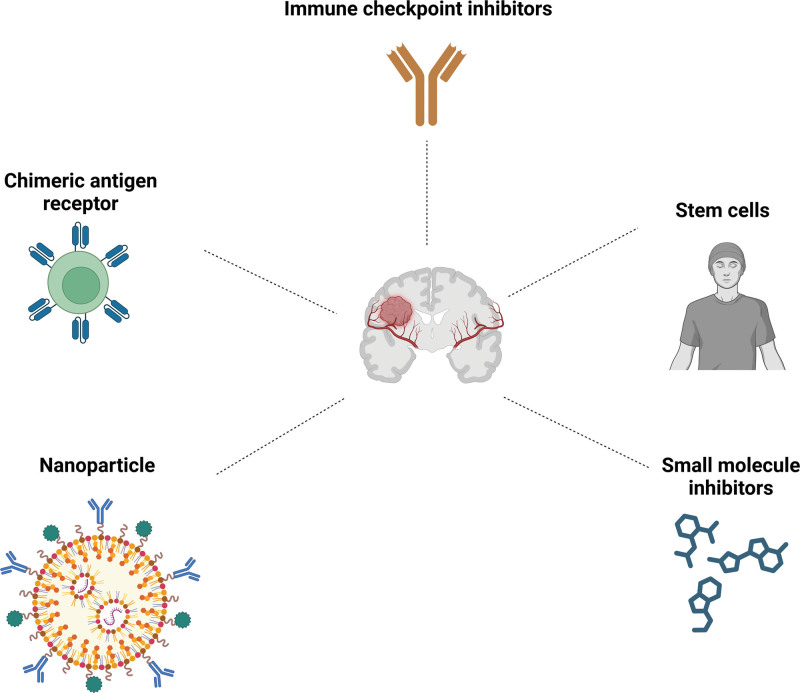
Integrated therapeutic approaches for GBM. A range of novel strategies has been established to address GBM. These include the utilization of immune checkpoint inhibitors to alleviate immunosuppression, the application of chimeric antigen receptor therapies for targeted tumor destruction, the use of nanoparticles to facilitate drug delivery across the blood–brain barrier, the development of small molecules aimed at specific signaling pathways, the employment of stem cells as therapeutic carriers, and the implementation of combination therapies to augment treatment efficacy.

In this review, we provide a comprehensive overview of the features of the inhibitory TME and its mechanisms of interaction with GBM tumor cells (Fig. 1). This analysis highlights the pivotal role of the microenvironment in contributing to the therapeutic resistance observed in GBM, thereby offering novel insights into the pathophysiological processes underlying this malignancy. Additionally, we advocate for the development of advanced therapeutic approaches targeting the inhibitory TME (Fig. 2). We examine the potential integration of novel technologies, including nanoparticles and chimeric antigen receptor (CAR) therapies, in the treatment of GBM. Furthermore, we suggest therapeutic strategies that focus on the modulation of the microenvironment, which may contribute to the advancement of next-generation therapeutics for GBM.

## 2. TME

The term TME denotes the intricate interplay of cells and molecules that support the growth and evolution of GBM. TME is comprised of an intricate network of cancerous cells, stromal cells, immune cells, and various bioactive molecules that are released by these cells.^[20,21]^ TME not only serves as a conducive medium for the growth and dissemination of GBM but also facilitates the expression of the aggressive characteristics of GBM through reciprocal communication between the tumor and its surrounding components.^[22]^ Particularly, the stromal cells within the TME have the capability to release a diverse array of growth factors, extracellular matrix components, and metabolites, which serve to facilitate the proliferation, invasion, and angiogenesis of GBM. Furthermore, immune cells present in the TME, including tumor-associated macrophages (TAMs) and myeloid-derived suppressor cells (MDSCs), among others, can contribute to the evasion of immune response by the tumor and confer resistance to therapeutic interventions through their interactions with tumor cells.^[23–25]^ There is also emerging evidence supporting the targeting of the TME for the treatment of GBM, demonstrating potential therapeutic benefits.^[26–28]^

## 3. Suppressive immune microenvironment

### 3.1. TAMs

In gliomas, particularly in the case of GBM, TAMs represent the predominant stromal cell population, comprising approximately 30% to 50% of the total cell population.^[25,29]^ TAMs are composed of bone marrow-derived macrophages and resident microglia.^[30]^ Despite carrying out comparable roles, the present research indicates that macrophages and microglia originate from distinct sources within the TME.^[31]^ Microglia serve as the primary intrinsic immune cells within the skull, originating from primitive myeloid progenitors during the initial stages of embryonic development. These progenitor cells retain the capacity for self-renewal following maturation within the cranium, thereby ensuring the maintenance of their population within this region.^[31,32]^ Microglia are significantly involved in maintaining the homeostasis of brain tissue and conducting immune surveillance in normal physiological circumstances. In contrast, macrophages originate from monocytes derived from bone marrow hematopoietic stem cells, which infiltrate blood vessels to travel to tumor tissue and undergo differentiation into macrophages.^[33]^ However, distinguishing between these 2 groups is challenging due to the absence of distinct cell surface markers.^[34]^ In the TME, alterations in the presentation of surface markers also pose challenges in distinguishing between the 2 groups.^[35]^ Recently, several sophisticated technologies such as single-cell sequencing, cell fate mapping, and microscopic imaging have broadened our comprehension of these 2 cellular populations.^[36–38]^ Further investigation is needed to understand the impact of microglia/macrophage composition and variations on the interaction network within the TME.

In a dichotomous model, TAMs are categorically divided into pro-inflammatory (M1) and pro-cancer (M2) phenotypes according to their functional characteristics. Typically, the M1 phenotype is evident in the initial phases of tumor formation and demonstrates anti-tumor effects, while the transition from M1 to M2 promotes the advancement of the tumor.^[39]^ It is important to acknowledge that the constraints of this dichotomous model are becoming evident, as there is growing evidence suggesting that the characteristics of TAMs are constantly changing and adaptable. In intricate TMEs, where both M1 and M2 macrophage types are often intertwined, the dynamics of the TME lead to the emergence of diverse TAM phenotypes.^[40]^ New subpopulations of TAMs have been identified using recent single-cell techniques,^[41,42]^ which represents a new individualized therapeutic strategy for addressing various states of TAMs.

A growing body of research is dedicated to examining the intricate interplay between TAMs and GBM. This interaction encompasses not only the growth, invasion, and metastasis of tumor cells but also the modulation of the host’s immune response. Specifically, TAMs can promote angiogenesis and alter the extracellular matrix for parenchyma invasion and tumor spread of GBM through multiple mechanisms,^[43–45]^ including the secretion of soluble mediators, direct interaction between cells, and the release of exosomes (reviewed in Broekman et al^[20]^). Furthermore, TAMs disrupt immune surveillance through various mechanisms, including the secretion of immunosuppressive factors (e.g., IL-6, transforming growth factor beta [TGF-β], IL-10),^[31]^ expression of CD47 molecules (“Don’t eat me” signals),^[46]^ and the alteration of immune checkpoint molecules (e.g., cytotoxic T lymphocyte associated antigen 4 [CTLA-4], programmed cell death protein 1 [PD-1]) to deactivate T-cell activity,^[47,48]^ thereby aiding GBM in evading immune system attacks.

TAMs are significantly involved in the progression of GBM, and manipulating TAM function may offer novel therapeutic approaches for GBM treatment. Preclinical studies have identified several promising strategies, including inhibiting TAM recruitment, enhancing TAM depletion, and reprogramming TAMs into M1 phenotypes, which exhibit anti-tumor effects.^[49–51]^ However, it is worth noting that the intricate nature of TAMs within the TME hinders the effectiveness of individual treatments, and focusing solely on TAMs may not be adequate for the complete eradication of tumors.^[52,53]^ Most current targeted therapies focus on modulating the activity and characteristics of TAMs rather than directly targeting TAMs themselves. Additionally, the potential for drug interactions with other cell types may reduce the efficacy of alternative treatment approaches, such as ICIs. This underscores the necessity of utilizing combination therapy. For instance, in a glioma model, the use of a colony-stimulating factor-1 receptor inhibitor in conjunction with radiotherapy demonstrated notable benefits in suppressing tumor recurrence compared to individual treatments.^[54]^ Likewise, the co-administration of IL-12-expressing lyssaviruses with 2 ICIs resulted in a notable extension of the survival of mice with GBM through the augmentation of M1-like TAMs and the enhancement of effector T-cell function.^[55]^ Clinical trials are currently being initiated to explore combination strategies for the treatment of GBM, which may potentially offer novel therapeutic options for patients.^[31]^

### 3.2. MDSCs

MDSCs are a subset of immunosuppressive cells originating from the bone marrow. In healthy circumstances, myeloid cells undergo rapid differentiation and migration to various peripheral organs to carry out their immune functions. However, in pathological states such as chronic inflammation and cancer, myeloid progenitor cells and immature myeloid cells are hindered from progressing to maturity, leading to their accumulation at various stages of differentiation and eventual development into MDSCs.^[56]^ Based on their phenotype and morphology, MDSCs can be categorized into various types, such as granulocytes or polymorphonuclear cells, monocytes (M-MDSCs), and early MDSCs.^[57]^ In various types of tumors, the presence of MDSCs is linked to unfavorable prognosis and reduced response to treatment. In the case of GBM, there is a consistent elevation in the frequency of MDSCs, which have been observed to accumulate at the tumor site, as well as in the spleen and peripheral blood.^[58–61]^ Cytokines, transcription factors, chemokines, and exosomes released by tumor cells have been shown to play a role in the expansion and recruitment of MDSCs, facilitating the translocation of MDSCs into the TME (reviewed in Lakshmanachetty et al^[62]^).

While the specific type of MDSCs prevalent in the TME remains to be determined, a growing body of evidence indicates that MDSCs recruited to the TME utilize various mechanisms to exert immunosuppressive effects. These effects are linked to the inhibition of the activity of a broad spectrum of immune cells, including T cells, natural killer (NK) cells, and dendritic cells. Furthermore, MDSCs play a role in recruiting other immunosuppressive cells, such as regulatory T cells (Tregs), regulatory B cells, and M2 macrophages. This extensive immunosuppression contributes to the progression of GBM.^[63]^ Recent research has indicated that aside from their role in suppressing the immune system, MDSCs facilitate the advancement of tumors through non-immune pathways, including the modulation of angiogenesis and stromal degradation.^[64]^

Due to the immunosuppressive role of MDSCs, researchers have increasingly focused on the potential of targeting MDSCs as a therapeutic strategy for GBM in recent times. Numerous strategies have been devised to target MSDCs in GBM. For instance, the use of low-dose, metronomic capecitabine in conjunction with bevacizumab therapy has been shown to alleviate immunosuppression in the TME by reducing the population of circulating MDSCs.^[65]^ Recent research has also shown that bavituximab, a monoclonal antibody possessing anti-angiogenic characteristics, effectively acts on MDSCs within the TME and exhibits efficacy in treating newly diagnosed GBM.^[66]^ Approaches aimed at restraining the proliferation and recruitment of MSDCs have exhibited promising therapeutic implications. For instance, Takacs et al revealed that the migration of immunosuppressive M-MDSCs to the TME relied on CCL2 and CCL7, and concurrent targeting of CCL2 and CCL7 resulted in diminished MDSCs.^[67]^ Similarly, a nanoparticle-based CXCL12/CXCR4 pathway therapy attenuates the infiltration of M-MDSCs into the TME. When combined with radiotherapy, this treatment has led to prolonged survival in mice with GBM.^[68]^ In summary, the strategic targeting of MDSCs to restructure the functional TME represents a significant approach in the treatment of GBM. However, additional clinical investigations are required to comprehensively assess the feasibility and therapeutic potential of targeting MDSCs in this context.

### 3.3. Neutrophils: a dual role

Neutrophils exhibit a prompt response to infection and inflammation within the organism and were initially characterized as cellular components that combat pathogenic infections. The prevailing perspective suggests that chronic inflammation at the site of the tumor leads to significant recruitment of neutrophils. Within the TME, neutrophils exhibit a multifaceted role. On one hand, they facilitate tumor growth through various mechanisms, while on the other hand, they are implicated in diverse anti-tumor effects, indicating the heterogeneous and functionally diverse nature of neutrophils.^[69,70]^ In a manner akin to the categorization of macrophage polarization, the categorization of N1 and N2 was implemented to delineate neutrophils with anti-tumor and pro-tumor capabilities, respectively, predicated on their functional distinctions.^[71]^ These 2 categorizations may indicate distinct functional conditions of neutrophils within tumors; however, additional research is required to differentiate between these 2 cellular states.

While the investigation into the involvement of neutrophils in GBM is still in its early stages, several studies provide evidence for a potential contribution of neutrophils to the development of GBM. As far back as 1999, a research study demonstrated a connection between the grade of glioma and the rate of neutrophil infiltration, indicating a greater incidence of neutrophil infiltration in high-grade GBM.^[72]^ Additionally, Liang and colleagues’ investigation into the infiltration of neutrophils not only demonstrated a correlation with glioma grade and tumor progression but also with the development of resistance to anti- vascular endothelial growth factor (VEGF) therapy in patients with GBM.^[73]^ Rahbar et al conducted a study that emphasized the prognostic significance of neutrophil activation in the progression of disease among patients with GBM. Their findings indicated that patients exhibiting heightened neutrophil activity experienced a less favorable prognosis.^[74]^ A recent investigation examined the impact of specific microenvironments and tumor types on the characteristics of neutrophils. The study revealed that the microenvironment of brain tumors exhibited a greater presence of neutrophils and displayed a unique pro-inflammatory phenotype in comparison to neutrophils found in peripheral areas.^[75]^ Significantly, brain tumor neutrophils exhibit prolonged survival compared to peripheral neutrophils of similar characteristics, both through direct interaction and via soluble cytokines. These findings indicate that the microenvironment plays a crucial role in inducing changes in the phenotype and function of neutrophils and that neutrophils entering the brain undergo transcriptional alterations to acclimate to distinct microenvironments.^[75]^ It is noteworthy that there is a bidirectional crosstalk between glioma cells and neutrophils. The cytokine environment in GBM patients plays a role in recruiting neutrophils and prolonging their lifespan. Conversely, neutrophils in close proximity to the tumor enhance the production of immunosuppressive agents, such as reactive oxygen species, to sustain a persistent state of inflammation in the TME.^[76]^ A mechanistic investigation indicates that tumor cells experience ferroptosis when neutrophils penetrate the tumor stroma, leading to tumor necrosis in the progression of GBM.^[77]^

Numerous recent preclinical investigations have explored the potential of directing therapeutic interventions towards neutrophils in the context of treating GBM. One such example is the examination of Dapsone, which has been found to modulate neutrophil migration and chemotaxis and to demonstrate antitumor effects.^[78]^ Several treatments are currently in development that utilize the chemotactic properties of neutrophils to effectively target GBM cells. One such example is the utilization of drug delivery systems based on neutrophil membranes, which demonstrate high permeability through the BBB and notably enhance their ability to combat GBM.^[79]^ The neutrophil-based emerging CAR platform specifically delivers microenvironment-responsive nanomedicines and exhibits superior anti-GBM activity.^[80]^ However, as a result of the intricate interplay among the diverse cellular constituents within the TME, there is a compensatory recruitment of an alternative cell population when targeting a single-cell population alone.^[81]^ A thorough understanding of the intricate intercellular communication within the TME and the design of combination therapies that can effectively target multiple cell types are essential.

### 3.4. Tregs

Tregs are a class of T cell subpopulations with significant suppressive effects.^[82]^ Tregs are scarcely present in healthy brain tissue, but they are identifiable in 48% of GBM, indicating a higher prevalence of Tregs in high-grade gliomas.^[83]^ Furthermore, research has revealed that the percentage of Tregs present in tumor tissue is elevated in patients with GBM at 38.5%, compared to the peripheral blood of matched patients at 11.9%. Notably, the percentage of peripheral Tregs decreases in GBM patients following tumor resection and increases again upon recurrence, indicating a potential correlation between the proportion of Tregs and tumor burden.^[84]^ Comparable findings also indicated a notable elevation in the proportion of Tregs within tumor lesions among individuals diagnosed with GBM.^[85]^ Several studies have discussed the prognostic significance of Tregs in GBM. Wiencke et al found that higher levels of Tregs were associated with poorer survival rates.^[86]^ Another study demonstrated that a reduction in the proportion of Tregs in the peripheral blood following DC vaccination was linked to extended survival in patients with GBM.^[87]^

Understanding the mechanisms involved in the delicate balance of Treg migration and recruitment to GBM tumor tissue may be key to unlocking the immunosuppressive function of Tregs. The findings of Jordan et al indicate that the secretion of CCL2 and CCL22 by tumor cells plays a significant role in recruiting Tregs.^[88]^ Similarly, a separate investigation validated the high expression of the CCR4 chemokine receptor in Tregs within GBM, thereby emphasizing the significant involvement of chemokines in the attraction of Tregs.^[89]^ The process of CCL2 production within the TME was investigated by Chang AL et al. Their findings indicated that cytokines released by tumor cells stimulated macrophages and microglia to generate CCL2, which subsequently recruited CCR4-expressing Tregs. Significantly, the study demonstrated that the use of small-molecule inhibitors targeting CCR4 reduced the accumulation of Tregs.^[90]^ Furthermore, Crane CA noted that the supernatants of GBM cells transiently facilitated the differentiation of T cells into Tregs, as indicated by the expression of TGF-β and FOXP3, implying an alternative pathway for recruiting Tregs in the TME.^[84]^

It is widely acknowledged that Tregs create and sustain an immunosuppressive environment through a range of mechanisms.^[91]^ In malignant gliomas, glioma cells have the capacity to stimulate the proliferation of Tregs through non-dependent mechanisms, including the release of cytokines such as TGF-β and IL-10. Furthermore, glioma cells also contribute to the accumulation of Tregs by activating other elements of the microenvironment, such as MDSCs and TAMs, through the secretion of immunosuppressive factors.^[92]^ Conversely, Tregs also create an environment of immunosuppression by suppressing the activity of other immune cells, including T cells and NK cells.^[92]^ However, whether Tregs have a direct interaction on GBM still requires further investigation.

CD25 is a well-established surface marker of Tregs, and recent research indicates that depletion of CD25 leads to elevated levels of activated CD4 T cells and an increased abundance of NK cells. Notably, CD25 depletion has been found to enhance the survival of mice with GBM following radiotherapy.^[93]^ Additional research has demonstrated that Tregs play a significant role in limiting the effectiveness of VEGF blockade therapy. It has been observed that the administration of an anti-CD25 antibody to block Tregs prior to VEGF blockade therapy can restore the anti-tumor responsiveness mediated by anti-VEGFR2.^[94]^ Therapeutic antibodies targeting CD25 have been utilized in clinical trials and have shown manageable levels of toxicity.^[95]^ Given that CD25 serves as a receptor subunit for IL-2 and that inhibiting CD25 can disrupt IL-2 signaling in effector T cells, Solomon et al devised a new anti-CD25 antibody (RG6292) with the aim of preserving IL-2 levels. Their findings indicated that this antibody reduced the suppressive function of Tregs and promoted T-cell activation in homozygous humanized mice.^[96]^ A clinical trial is currently in progress to evaluate the safety and effectiveness of Tregs depletion therapy in individuals with solid tumors (NCT04158583). While additional assessment is necessary for GBM, these developments underscore the potential therapeutic value of targeting Tregs for GBM.

## 4. Hypoxic microenvironment

Hypoxia has been identified as a significant characteristic present in all solid tumors.^[97]^ The heightened metabolic activity of GBM and the extended distance for oxygen diffusion resulting from an abnormal vascular system are recognized as significant contributors to the hypoxic conditions within the GBM microenvironment.^[98]^ In GBM, hypoxia triggers the activation of numerous target genes, leading to the establishment of an intricate network of processes that influence the malignant characteristics of tumor cells by impacting crucial signaling pathways. For instance, hypoxia promotes tumor proliferation, angiogenesis, invasion, and metastatic potential.^[99–101]^ Hypoxia has been linked to resistance to both radiotherapy and chemotherapy.^[102–104]^ Significantly, the development of malignant characteristics driven by hypoxia is closely linked to a negative prognosis in patients with GBM.^[105,106]^ Furthermore, GBM stem-like cells are present in a limited quantity within GBMs. These cells demonstrate characteristics akin to stem cells, including self-renewal and invasive properties. They are believed to be the primary instigators of tumor initiation, expansion, and reoccurrence in GBM.^[107]^ The increased expression of hypoxia-inducible factor 1 (HIF-1) in hypoxic environments has been demonstrated to support the maintenance and amplification of GBM stem-like cells.^[98]^

In GBM, hypoxia is involved in immunosuppression. GBM is commonly characterized as a “cold tumor” due to its limited immune cell infiltration and low neoantigen levels.^[108]^ Despite the presence of immune cells such as T cells and NK cells in the hypoxic microenvironment, they are still susceptible to damage. Conversely, the functionality of certain suppressor immune cells, such as Tregs and M2 macrophages, is heightened in this hypoxic setting.^[109]^ This renders hypoxia the most crucial inhibitory microenvironment in GBM.

In preclinical studies, several approaches have been investigated to block the activity of HIF-1, including manipulating HIF-1 protein synthesis, facilitating HIF-1 degradation, and reducing HIF-1 mRNA expression. These approaches have been tried and shown to ameliorate the malignant phenotype of tumor cells (reviewed in Domènech et al^[110]^). While additional research is needed to explore greater specificity and more effective approaches, targeting the hypoxic microenvironment could be a promising strategy to impede the progression of GBM, given its significant role as a key driver of GBM advancement.

## 5. Metabolic microenvironment: focus on glycolytic metabolism

GBM is distinguished by its rapid proliferation and extensive infiltration, leading to the development of nutrient deficiencies within the microenvironment. In order to obtain sufficient nutrients, the metabolic pattern of tumor cells is altered, and aerobic glycolysis (Warburg effect) is an important feature of abnormal metabolism in tumor cells.^[111]^ To meet the elevated metabolic requirements of GBM, there is an increase in the expression of glucose transporters and glycolytic enzymes essential for glycolysis. Research has demonstrated that GLUT1 is significantly upregulated in GBM, and its expression levels correlate with the relative glucose concentration in the TME.^[112]^ Furthermore, there was an increase in the expression of glycolytic enzymes in GBM, which was correlated with a decrease in the overall survival rate of patients.^[113,114]^ In contrast, the elimination or suppression of glycolytic enzymes resulted in a survival advantage in xenograft mice, indicating the significant involvement of glycolysis in the growth of GBM.^[113,115]^

Increased glycolytic metabolism is not exclusive to cancer cells within the TME. This significant metabolic characteristic can provide energy support for various cells, including immune cells.^[114]^ This increased glycolysis plays a crucial role in providing metabolic support to immune cells, particularly T-cells, enabling them to undergo rapid proliferation and transition into an activated state.^[116]^ On the contrary, swift depletion of glucose in glycolytic metabolism results in an elevated need for oxygen in the local environment, causing tissue hypoxia. This condition contributes to the shaping of an inhibitory immune microenvironment. Furthermore, lactate, which is the final byproduct of glycolytic metabolism, accumulates in the microenvironment and directly impacts immune cells. This impact includes the inhibition of anti-tumor immune cells, such as T cells and NK cells, as well as the promotion of immune-suppressor cell populations, such as MDSCs.^[16]^ Additional research focusing on the manipulation of glycolysis to reverse the immunosuppressive effects caused by heightened glycolysis is a promising area of interest and offers a compelling approach to altering responsiveness to immunotherapy.

## 6. Novel therapeutic strategies to overcome GBM

### 6.1. ICIs

ICIs are a category of pharmaceutical agents that augment the body’s natural anti-tumor immune response by obstructing immunosuppressive signals, thereby stimulating or facilitating immune cell activation and mobilization.^[117]^ ICI has demonstrated notable anti-tumor efficacy across various tumor categories and is currently being utilized in conjunction with other pharmaceutical agents to augment its clinical utility.^[118]^

In GBM, frequently utilized ICIs consist of the PD-1 antibody and the CTLA-4 antibody. In recent years, numerous clinical trials have been conducted to assess the efficacy of ICIs in the treatment of GBM. In a phase 3 clinical trial (CheckMate 143), 369 patients diagnosed with GBM who experienced a 1st relapse following standard radiotherapy and temozolomide (TMZ) treatment were randomly allocated in a 1:1 ratio to receive either Nivolumab, an anti-PD-1 therapy, or Bevacizumab. The findings indicated that the median overall survival in the Nivolumab group was comparable to that of the Bevacizumab group, with respective durations of 9.8 months and 10 months.^[14]^ Similarly, in an open-label phase 3 clinical trial (CheckMate 498), Nivolumab in combination with radiotherapy did not improve survival in patients with newly diagnosed unmethylated MGMT promoter GBM compared with TMZ in combination with radiotherapy.^[119]^ In a separate phase 3 clinical study involving patients with newly diagnosed GBM and methylated MGMT promoter (CheckMate 548), researchers noted that the survival advantage of the Nivolumab + radiotherapy + TMZ combination was not found to be significantly better than that of the radiotherapy + TMZ + placebo combination.^[120]^

Although ICIs have not demonstrated significant efficacy in unselected GBM patients, multiple studies have indicated that GBM patients with distinct molecular profiles exhibit improved imaging response and survival outcomes following ICIs treatment.^[121,122]^ Numerous studies have also examined the possibility of using ICIs in neoadjuvant immunotherapy for GBM. For instance, a study involving 35 patients with recurrent, surgically removable GBM found that those who underwent PD-1 therapy before surgical resection and continued immunotherapy after the operation experienced extended overall survival compared to patients who received PD-1 therapy solely after the surgery.^[123]^ A similar clinical study also noted that certain patients with GBM who received ICIs as adjuvant therapy exhibited improved survival outcomes.^[124]^

Based on this information, it is essential to consider the distinct molecular features of patients when considering the use of ICIs for GBM treatment in the future. Additionally, it is crucial to establish early indicators that can predict the response to ICIs therapy. This will facilitate the identification of patient populations that are likely to experience clinical benefits from ICI treatment.

### 6.2. CAR

The concept of CAR originated in 1989, when Gross G and colleagues suggested that equipping T cells with a chimeric receptor could be a significant approach in combating tumors. Following years of advancement, CAR-T cell therapy has demonstrated notable efficacy in treating various malignant tumors, particularly hematological tumors, and is being explored for potential application in solid tumors.^[125]^

In GBM, promising preclinical outcomes have been observed with CAR-T cell therapies targeting various antigens such as IL13Rα2, EGFRvIII, HER2, EphA2, and GD2.^[126]^ These targets have also undergone assessment in clinical trials and demonstrate significant potential for the treatment of GBM.^[127]^ In an instance, during a phase I clinical trial focused on IL13Rα2, the administration of multiple infusions of CAR-T cells through 2 intracranial routes demonstrated that the CAR-T therapy facilitated regression of intracranial tumors and resulted in temporary complete remission in patients with recurrent GBM. Additionally, the treatment-related toxicities were found to be manageable.^[128]^ In a separate phase I clinical trial focused on EGFRvIII, a group of 10 patients experiencing recurrent GBM received treatment with CAR-T cells. These cells were found to be delivered to the tumor site following intravenous administration and demonstrated impact on the TME. Specifically, certain immunosuppressive molecules were observed to be increased after CAR-T infusion, highlighting the constraints posed by the immunosuppressive microenvironment on CAR-T cell therapy.^[129]^ A clinical trial testing the feasibility and safety of allogeneic CAR-T cell therapy for the treatment of recurrent unresectable GBM has shown that a transient anti-tumor effect was achieved in some patients.^[130]^ Another research investigation assessed the efficacy of CAR-modified virus-specific T-cell therapy in treating individuals with HER2-positive GBM. The study revealed that out of 16 patients evaluated, 1 experienced partial remission while 7 exhibited stable disease, indicating the promising clinical utility of this therapeutic approach.^[131]^

NK cells are a type of innate lymphocyte that possess distinct biological capabilities. Unlike T cells, NK cells do not necessitate prior sensitization for the recognition and effective elimination of tumor cells.^[132]^ Creating CAR utilizing NK cells as the effector cells not only maintains the inherent anti-tumor reactivity of NK cells but also enables NK cells to selectively home in on tumors. In recent studies, there is increasing evidence to suggest that CAR-NK cell therapy has emerged as a promising immunotherapy approach for the treatment of GBM.^[133]^ Comparable to the targets employed in CAR-T therapies, CAR-NK therapies directed at antigens such as HER2, EGFRvIII, and GD2 have exhibited potent anti-GBM activity in preclinical investigations.^[134–136]^ The research conducted by Wang and colleagues introduces a multifunctional approach involving CAR-NK cells, which has been demonstrated to augment the infiltration of NK cells and facilitate the eradication of tumors.^[137]^ A current clinical trial is being conducted to assess the safety and tolerance of intracranial administration of NK-92/5.28.zCAR NK cells in individuals with recurrent HER2-positive GBM (NCT03383978).

### 6.3. Nanoparticle

The primary factor restricting effective treatment of GBM is the restricted permeability of drugs through the BBB.^[138]^ There is a pressing requirement for a technology that enhances the effectiveness of current medications and facilitates the transportation of chemotherapeutic agents across the BBB. The utilization of nanocarriers in drug delivery represents a viable strategy due to their capacity to transport therapeutic agents (such as anti-cancer drugs, proteins, nucleic acids, etc) and shield these compounds from premature degradation or release.^[139]^ A synthetic protein nanoparticle based on polymerized human serum albumin has demonstrated the ability to traverse the BBB for targeted delivery of anticancer medications to GBM.^[140]^ Similarly, an synthetic protein nanoparticle therapy encapsulating a CXCL2/CXCR4 pathway antagonist (AMD3100) in combination with radiotherapy achieves long-term survival and triggers a beneficial immune microenvironment in hormonal mice.^[68]^ Cisplatin-loaded nanoparticles were capable of utilizing MR-guided focused ultrasound to traverse the BBB and effectively suppress the growth of GBM.^[141]^

Numerous clinical trials have been registered on ClinicalTrials.gov to assess the application of nanoparticles in drug delivery for the treatment of GBM (NCT04881032, NCT03020017, NCT00734682). However, the intricate process of nanoparticle preparation and characterization, as well as the assessment of biodistribution and safety considerations, presents significant challenges for the utilization of nanoparticles in the treatment of GBM.^[142]^ There is a need for further research to develop suitable nanocarriers for efficient drug delivery and to explore different routes for delivery of therapeutic agents to the central nervous system.

### 6.4. Small molecule inhibitors

TMZ is currently the only 1st-line chemotherapeutic agent for GBM; however, his chemotherapy regimen is susceptible to systemic adverse effects, initial resistance to TMZ, or the development of resistance during treatment, all of which restrict its effectiveness in combating tumors.^[143]^ Over the past decades, multilevel solutions have been investigated, such as the development of novel small molecule drugs. Currently, several small molecule inhibitors have been extensively tested in GBM patients, mainly targeting the major dysregulated pathways in GBM, including receptor tyrosine kinases, the PI3K/AKT/mTOR pathway, the p53 pathway, and the RB pathway.^[144]^

The ineffectiveness of most small molecule inhibitors in the treatment of GBM can largely be ascribed to the insufficient specificity of the current therapeutic targets. Many of these targets are also implicated in peripheral tumors, leading to concurrent targeting that diminishes therapeutic efficacy.^[144]^ Most small molecule therapies in current clinical studies for GBM have a limited duration of exposure, thereby constraining their effectiveness.^[145]^ Insufficient permeation of the BBB is a significant factor that restricts the clinical application of small molecule inhibitors. Despite their limitations, small molecule inhibitors will continue to be the primary focus for the development of therapeutic drugs for GBM in the future. This is due to their relatively uncomplicated structure and cost-effective synthesis. As our understanding of the pathogenesis of GBM and molecular markers advances, more targeted and BBB-penetrating small molecule compounds will remain the predominant approach for GBM drug development.

### 6.5. Stem cells

Stem cells are a class of cells characterized by their ability to self-renew and differentiate into various cell types. Subsets of stem cells, such as mesenchymal stem cells (MSC), hematopoietic stem cells, and neural stem cells (NSC), possess the capability to migrate to tumor sites. This migration enables the delivery of diverse therapeutic agents, such as tumor-destroying viruses, cytokines, antibodies, and nanoparticles, thereby enhancing the efficacy of these treatments.^[146]^ In recent years, stem cells have emerged as a novel strategy for the treatment of GBM. For instance, a hybrid MSC/nanosphere system has been developed to enhance drug retention at the tumor site and demonstrate substantial anti-tumor effects.^[147]^ The utilization of NSC membrane for the encapsulation and transportation of lysosomal viruses demonstrates outstanding abilities to traverse the BBB and target tumors.^[148]^

Two active phase I clinical trials have been implemented to assess the efficacy of genetically modified MSCs in treating GBM (NCT03896568, NCT04657315). Additionally, several completed phase I trials have utilized genetically engineered NSCs in patients with high-grade gliomas (NCT01172964, NCT02015819). Despite remaining ethical and financial considerations, the emergence of these treatments undoubtedly presents promising new therapeutic prospects for GBM.

Another field of interest pertains to the potential of utilizing exosomes derived from stem cells for therapeutic purposes in the context of GBM.^[149]^ Research has indicated that MSC and NSC possess the ability to produce exosomes.^[150]^ MSC-derived exosomes have been shown to improve tumor progression by releasing therapeutic small molecules, including miRNA-584.^[151]^ Additionally, these exosomes possess the capability to penetrate the BBB and offer benefits such as minimal immune response and tumor homing.^[152,153]^ Utilizing these characteristics, researchers have employed exosomes as delivery vehicles for the inhibition of tumors through the transportation of proteins, miRNAs, and chemicals.^[154,155]^

## 7. Conclusions and directions

The TME is a complex ecological network within GBM. Within the TME, various inhibitory microenvironments, such as suppressor immune cells, hypoxia, and nutrient deficiencies, closely crosstalk with cancer cells and impact the biological behavior and response to treatment of GBM. A comprehensive understanding of these crosstalks is crucial for the advancement of novel therapeutic approaches. This review offers valuable insights into the crosstalk between multiple inhibitory microenvironments and GBM and explores the potential of targeting these microenvironments for therapeutic purposes. Nevertheless, it is important to acknowledge that the development of therapeutic agents targeting these microenvironments necessitates extensive experimental and clinical testing.

Immunotherapy and molecularly targeted therapies are currently the predominant treatment modalities for GBM, offering advantages in delaying recurrence and enhancing survival rates. However, the clinical outcomes for GBM have not shown significant improvement due to its pronounced heterogeneity and ability to evade the immune system. Ongoing research is exploring alternative approaches for GBM, including the development of nanoparticles with enhanced permeability through the BBB and unique properties for targeting tumor cells. Additionally, CAR-based therapies represent a crucial advancement towards personalized GBM treatment. Given the intricate nature of the TME, the effectiveness of individual therapies is often insufficient, necessitating the development of strategies to combine these therapies to maximize efficacy.

## Author contributions

**Conceptualization**: Yi Wei, Yunjuan Zhang.

**Funding acquisition**: Yi Wei.

**Methodology**: Yi Wei, Yunjuan Zhang.

**Validation**: Yunjuan Zhang.

**Visualization**: Yuwei Shi.

**Writing – original draft**: Yi Wei.

**Writing – review & editing**: Hu Wang, Yinwu Shang, Bingwen Yu, Yunjuan Zhang.

## References

[1] Li M Chen F Yang Q. Biomaterial-based CRISPR/Cas9 delivery systems for tumor treatment. Biomater Res. 2024;28:0023. 38694229 10.34133/bmr.0023 PMC11062511

[2] Xiao M Tang Q Zeng S. Emerging biomaterials for tumor immunotherapy. Biomater Res. 2023;27:47. 37194085 10.1186/s40824-023-00369-8 PMC10189985

[3] Yang Q Li S Ou H. Exosome-based delivery strategies for tumor therapy: an update on modification, loading, and clinical application. J Nanobiotechnology. 2024;22:41. 38281957 10.1186/s12951-024-02298-7 PMC10823703

[4] Brodbelt A Greenberg D Winters T Williams M Vernon S Collins VP. Glioblastoma in England: 2007–2011. Eur J Cancer. 2015;51:533–42. 25661102 10.1016/j.ejca.2014.12.014

[5] Ostrom QT Price M Neff C. CBTRUS statistical report: primary brain and other central nervous system tumors diagnosed in the United States in 2016–2020. Neuro-Oncology. 2023;25(Supplement_4):iv1–iv99. 37793125 10.1093/neuonc/noad149 PMC10550277

[6] Stupp R Mason WP van den Bent MJ. Radiotherapy plus concomitant and adjuvant temozolomide for glioblastoma. N Engl J Med. 2005;352:987–96. 15758009 10.1056/NEJMoa043330

[7] Dewdney B Jenkins MR Best SA. From signalling pathways to targeted therapies: unravelling glioblastoma’s secrets and harnessing two decades of progress. Signal Transduct Target Ther. 2023;8:400. 37857607 10.1038/s41392-023-01637-8 PMC10587102

[8] Tan AC Ashley DM López GY Malinzak M Friedman HS Khasraw M. Management of glioblastoma: state of the art and future directions. CA Cancer J Clin. 2020;70:299–312. 32478924 10.3322/caac.21613

[9] Tykocki T Eltayeb M. Ten-year survival in glioblastoma. a systematic review. J Clin Neurosci. 2018;54:7–13. 29801989 10.1016/j.jocn.2018.05.002

[10] Qazi MA Vora P Venugopal C. Intratumoral heterogeneity: pathways to treatment resistance and relapse in human glioblastoma. Ann Oncol. 2017;28:1448–56. 28407030 10.1093/annonc/mdx169

[11] Da Ros M De Gregorio V Iorio AL. Glioblastoma chemoresistance: the double play by microenvironment and blood–brain barrier. Int J Mol Sci. 2018;19:2879. 30248992 10.3390/ijms19102879 PMC6213072

[12] Ahmad A Khan P Rehman AU Batra SK Nasser MW. Immunotherapy: an emerging modality to checkmate brain metastasis. Mol Cancer. 2023;22:111. 37454123 10.1186/s12943-023-01818-7 PMC10349473

[13] Kirkwood JM Butterfield LH Tarhini AA Zarour H Kalinski P Ferrone S. Immunotherapy of cancer in 2012. CA Cancer J Clin. 2012;62:309–35. 22576456 10.3322/caac.20132 PMC3445708

[14] Reardon DA Brandes AA Omuro A. Effect of nivolumab vs bevacizumab in patients with recurrent glioblastoma: the checkmate 143 phase 3 randomized clinical trial. JAMA Oncol. 2020;6:1003–10. 32437507 10.1001/jamaoncol.2020.1024 PMC7243167

[15] Zhou Y Cheng L Liu L Li X. NK cells are never alone: crosstalk and communication in tumour microenvironments. Mol Cancer. 2023;22:34. 36797782 10.1186/s12943-023-01737-7 PMC9933398

[16] Choudhary N Osorio RC Oh JY Aghi MK. Metabolic barriers to glioblastoma immunotherapy. Cancers (Basel). 2023;15:1519. 36900311 10.3390/cancers15051519 PMC10000693

[17] Perrin SL Samuel MS Koszyca B. Glioblastoma heterogeneity and the tumour microenvironment: implications for preclinical research and development of new treatments. Biochem Soc Trans. 2019;47:625–38. 30902924 10.1042/BST20180444

[18] Zhong X He X Wang Y. Warburg effect in colorectal cancer: the emerging roles in tumor microenvironment and therapeutic implications. J Hematol Oncol. 2022;15:160. 36319992 10.1186/s13045-022-01358-5 PMC9628128

[19] Habashy KJ Mansour R Moussalem C Sawaya R Massaad MJ. Challenges in glioblastoma immunotherapy: mechanisms of resistance and therapeutic approaches to overcome them. Br J Cancer. 2022;127:976–87. 35662275 10.1038/s41416-022-01864-w PMC9470562

[20] Broekman ML Maas SLN Abels ER Mempel TR Krichevsky AM Breakefield XO. Multidimensional communication in the microenvirons of glioblastoma. Nat Rev Neurol. 2018;14:482–95. 29985475 10.1038/s41582-018-0025-8 PMC6425928

[21] Dapash M Hou D Castro B Lee-Chang C Lesniak MS. The interplay between glioblastoma and its microenvironment. Cells. 2021;10:2257. 34571905 10.3390/cells10092257 PMC8469987

[22] Pang L Khan F Heimberger AB Chen P. Mechanism and therapeutic potential of tumor-immune symbiosis in glioblastoma. Trends Cancer. 2022;8:839–54. 35624002 10.1016/j.trecan.2022.04.010 PMC9492629

[23] Jackson C Ruzevick J Phallen J Belcaid Z Lim M. Challenges in immunotherapy presented by the glioblastoma multiforme microenvironment. Clin Dev Immunol. 2011;2011:732413. 22190972 10.1155/2011/732413 PMC3235820

[24] Hernández A Domènech M Muñoz-Mármol AM Carrato C Balana C. Glioblastoma: relationship between metabolism and immunosuppressive microenvironment. Cells. 2021;10:3529. 34944036 10.3390/cells10123529 PMC8700075

[25] Xuan W Lesniak MS James CD Heimberger AB Chen P. Context-dependent glioblastoma–macrophage/microglia symbiosis and associated mechanisms. Trends Immunol. 2021;42:280–92. 33663953 10.1016/j.it.2021.02.004 PMC8005482

[26] Wang G Zhang Z Zhong K. CXCL11-armed oncolytic adenoviruses enhance CAR-T cell therapeutic efficacy and reprogram tumor microenvironment in glioblastoma. Mol Ther. 2023;31:134–53. 36056553 10.1016/j.ymthe.2022.08.021 PMC9840126

[27] Berger G Knelson EH Jimenez-Macias JL. STING activation promotes robust immune response and NK cell-mediated tumor regression in glioblastoma models. Proc Natl Acad Sci USA. 2022;119:e2111003119. 35787058 10.1073/pnas.2111003119 PMC9282249

[28] Geraldo LH Xu Y Jacob L. SLIT2/ROBO signaling in tumor-associated microglia and macrophages drives glioblastoma immunosuppression and vascular dysmorphia. J Clin Invest. 2021;131:e141083. 34181595 10.1172/JCI141083 PMC8363292

[29] Gutmann DH Kettenmann H. Microglia/brain macrophages as central drivers of brain tumor pathobiology. Neuron. 2019;104:442–9. 31697921 10.1016/j.neuron.2019.08.028 PMC7288606

[30] Ricard C Tchoghandjian A Luche H. Phenotypic dynamics of microglial and monocyte-derived cells in glioblastoma-bearing mice. Sci Rep. 2016;6:26381. 27193333 10.1038/srep26381 PMC4872227

[31] Wang G Zhong K Wang Z. Tumor-associated microglia and macrophages in glioblastoma: from basic insights to therapeutic opportunities. Front Immunol. 2022;13:964898. 35967394 10.3389/fimmu.2022.964898 PMC9363573

[32] Ginhoux F Greter M Leboeuf M. Fate mapping analysis reveals that adult microglia derive from primitive macrophages. Science. 2010;330:841–5. 20966214 10.1126/science.1194637 PMC3719181

[33] Khan F Pang L Dunterman M Lesniak MS Heimberger AB Chen P. Macrophages and microglia in glioblastoma: heterogeneity, plasticity, and therapy. J Clin Invest. 2023;133:e163446. 36594466 10.1172/JCI163446 PMC9797335

[34] Chen Z Feng X Herting CJ. Cellular and molecular identity of tumor-associated macrophages in glioblastoma. Cancer Res. 2017;77:2266–78. 28235764 10.1158/0008-5472.CAN-16-2310 PMC5741820

[35] Müller A Brandenburg S Turkowski K Müller S Vajkoczy P. Resident microglia, and not peripheral macrophages, are the main source of brain tumor mononuclear cells. Int J Cancer. 2015;137:278–88. 25477239 10.1002/ijc.29379

[36] Pombo Antunes AR Scheyltjens I Lodi F. Single-cell profiling of myeloid cells in glioblastoma across species and disease stage reveals macrophage competition and specialization. Nat Neurosci. 2021;24:595–610. 33782623 10.1038/s41593-020-00789-y

[37] Sankowski R Böttcher C Masuda T. Mapping microglia states in the human brain through the integration of high-dimensional techniques. Nat Neurosci. 2019;22:2098–110. 31740814 10.1038/s41593-019-0532-y

[38] Chen Z Ross JL Hambardzumyan D. Intravital 2-photon imaging reveals distinct morphology and infiltrative properties of glioblastoma-associated macrophages. Proc Natl Acad Sci USA. 2019;116:14254–9. 31235603 10.1073/pnas.1902366116 PMC6628659

[39] Liu J Geng X Hou J Wu G. New insights into M1/M2 macrophages: key modulators in cancer progression. Cancer Cell Int. 2021;21:389. 34289846 10.1186/s12935-021-02089-2 PMC8296555

[40] Murray PJ Allen JE Biswas SK. Macrophage activation and polarization: nomenclature and experimental guidelines. Immunity. 2014;41:14–20. 25035950 10.1016/j.immuni.2014.06.008 PMC4123412

[41] Yeo AT Rawal S Delcuze B. Single-cell RNA sequencing reveals evolution of immune landscape during glioblastoma progression. Nat Immunol. 2022;23:971–84. 35624211 10.1038/s41590-022-01215-0 PMC9174057

[42] Ochocka N Segit P Walentynowicz KA. Single-cell RNA sequencing reveals functional heterogeneity of glioma-associated brain macrophages. Nat Commun. 2021;12:1151. 33608526 10.1038/s41467-021-21407-w PMC7895824

[43] Cui X Morales RT Qian W. Hacking macrophage-associated immunosuppression for regulating glioblastoma angiogenesis. Biomaterials. 2018;161:164–78. 29421553 10.1016/j.biomaterials.2018.01.053 PMC8059366

[44] Dello Russo C Lisi L Tentori L Navarra P Graziani G Combs CK. Exploiting microglial functions for the treatment of glioblastoma. Curr Cancer Drug Targets. 2017;17:267–81. 27528361 10.2174/1568009616666160813191240

[45] Brandenburg S Müller A Turkowski K. Resident microglia rather than peripheral macrophages promote vascularization in brain tumors and are source of alternative pro-angiogenic factors. Acta Neuropathol. 2016;131:365–78. 26718201 10.1007/s00401-015-1529-6

[46] Hutter G Theruvath J Graef CM. Microglia are effector cells of CD47-SIRPα antiphagocytic axis disruption against glioblastoma. Proc Natl Acad Sci USA. 2019;116:997–1006. 30602457 10.1073/pnas.1721434116 PMC6338872

[47] Dumas AA Pomella N Rosser G. Microglia promote glioblastoma via mTOR-mediated immunosuppression of the tumour microenvironment. EMBO J. 2020;39:e103790. 32567735 10.15252/embj.2019103790 PMC7396846

[48] Mei Y Wang X Zhang J. Siglec-9 acts as an immune-checkpoint molecule on macrophages in glioblastoma, restricting T-cell priming and immunotherapy response. Nat Cancer. 2023;4:1273–91. 37460871 10.1038/s43018-023-00598-9

[49] Takenaka MC Gabriely G Rothhammer V. Control of tumor-associated macrophages and T cells in glioblastoma via AHR and CD39. Nat Neurosci. 2019;22:729–40. 30962630 10.1038/s41593-019-0370-y PMC8052632

[50] Markovic DS Vinnakota K Chirasani S. Gliomas induce and exploit microglial MT1-MMP expression for tumor expansion. Proc Natl Acad Sci USA. 2009;106:12530–5. 19617536 10.1073/pnas.0804273106 PMC2718387

[51] Fujiwara Y Komohara Y Kudo R. Oleanolic acid inhibits macrophage differentiation into the M2 phenotype and glioblastoma cell proliferation by suppressing the activation of STAT3. Oncol Rep. 2011;26:1533–7. 21922144 10.3892/or.2011.1454

[52] Zhang L Jiang Y Zhang G Wei S. The diversity and dynamics of tumor-associated macrophages in recurrent glioblastoma. Front Immunol. 2023;14:1238233. 37731483 10.3389/fimmu.2023.1238233 PMC10507272

[53] Butowski N Colman H De Groot JF. Orally administered colony stimulating factor 1 receptor inhibitor PLX3397 in recurrent glioblastoma: an Ivy Foundation Early Phase Clinical Trials Consortium phase II study. Neuro Oncol. 2016;18:557–64. 26449250 10.1093/neuonc/nov245 PMC4799682

[54] Akkari L Bowman RL Tessier J. Dynamic changes in glioma macrophage populations after radiotherapy reveal CSF-1R inhibition as a strategy to overcome resistance. Sci Transl Med. 2020;12:eaaw7843. 32669424 10.1126/scitranslmed.aaw7843

[55] Saha D Martuza RL Rabkin SD. Macrophage polarization contributes to glioblastoma eradication by combination immunovirotherapy and immune checkpoint blockade. Cancer Cell. 2017;32:253–67.e5. 28810147 10.1016/j.ccell.2017.07.006 PMC5568814

[56] Wu Y Yi M Niu M Mei Q Wu K. Myeloid-derived suppressor cells: an emerging target for anticancer immunotherapy. Mol Cancer. 2022;21:184. 36163047 10.1186/s12943-022-01657-y PMC9513992

[57] Hao Z Li R Wang Y Li S Hong Z Han Z. Landscape of myeloid-derived suppressor cell in tumor immunotherapy. Biomarker Res. 2021;9:77. 10.1186/s40364-021-00333-5 PMC8543853 34689842

[58] Raychaudhuri B Rayman P Ireland J. Myeloid-derived suppressor cell accumulation and function in patients with newly diagnosed glioblastoma. Neuro-Oncology. 2011;13:591–9. 21636707 10.1093/neuonc/nor042 PMC3107102

[59] Gielen PR Schulte BM Kers-Rebel ED. Elevated levels of polymorphonuclear myeloid-derived suppressor cells in patients with glioblastoma highly express S100A8/9 and arginase and suppress T cell function. Neuro-Oncology. 2016;18:1253–64. 27006175 10.1093/neuonc/now034 PMC4998996

[60] Dubinski D Wölfer J Hasselblatt M. CD4+ T effector memory cell dysfunction is associated with the accumulation of granulocytic myeloid-derived suppressor cells in glioblastoma patients. Neuro-Oncology. 2016;18:807–18. 26578623 10.1093/neuonc/nov280 PMC4864257

[61] Alban TJ Alvarado AG Sorensen MD. Global immune fingerprinting in glioblastoma patient peripheral blood reveals immune-suppression signatures associated with prognosis. JCI Insight. 2018;3:e122264. 30385717 10.1172/jci.insight.122264 PMC6238746

[62] Lakshmanachetty S Cruz-Cruz J Hoffmeyer E Cole AP Mitra SS. New insights into the multifaceted role of myeloid-derived suppressor cells (MDSCs) in high-grade gliomas: from metabolic reprograming, immunosuppression, and therapeutic resistance to current strategies for targeting MDSCs. Cells. 2021;10:893. 33919732 10.3390/cells10040893 PMC8070707

[63] Richard SA. Explicating the pivotal pathogenic, diagnostic, and therapeutic biomarker potentials of myeloid-derived suppressor cells in glioblastoma. Dis Markers. 2020;2020:8844313. 33204365 10.1155/2020/8844313 PMC7657691

[64] Won WJ Deshane JS Leavenworth JW Oliva CR Griguer CE. Metabolic and functional reprogramming of myeloid-derived suppressor cells and their therapeutic control in glioblastoma. Cell Stress. 2019;3:47–65. 31225500 10.15698/cst2019.02.176 PMC6551710

[65] Peereboom DM Alban TJ Grabowski MM. Metronomic capecitabine as an immune modulator in glioblastoma patients reduces myeloid-derived suppressor cells. JCI Insight. 2019;4:e130748. 31600167 10.1172/jci.insight.130748 PMC6948860

[66] Ly KI Richardson LG Liu M. Bavituximab decreases immunosuppressive myeloid-derived suppressor cells in newly diagnosed glioblastoma patients. Clin Cancer Res. 2023;29:3017–25. 37327319 10.1158/1078-0432.CCR-23-0203

[67] Takacs GP Kreiger CJ Luo D. Glioma-derived CCL2 and CCL7 mediate migration of immune suppressive CCR2(+)/CX3CR1(+) M-MDSCs into the tumor microenvironment in a redundant manner. Front Immunol. 2022;13:993444. 36685592 10.3389/fimmu.2022.993444 PMC9854274

[68] Alghamri MS Banerjee K Mujeeb AA. Systemic delivery of an adjuvant CXCR4-CXCL12 signaling inhibitor encapsulated in synthetic protein nanoparticles for glioma immunotherapy. ACS Nano. 2022;16:8729–50. 35616289 10.1021/acsnano.1c07492 PMC9649873

[69] Jaillon S Ponzetta A Di Mitri D Santoni A Bonecchi R Mantovani A. Neutrophil diversity and plasticity in tumour progression and therapy. Nat Rev Cancer. 2020;20:485–503. 32694624 10.1038/s41568-020-0281-y

[70] Xiong S Dong L Cheng L. Neutrophils in cancer carcinogenesis and metastasis. J Hematol Oncol. 2021;14:173. 34674757 10.1186/s13045-021-01187-y PMC8529570

[71] Fridlender ZG Sun J Kim S. Polarization of tumor-associated neutrophil phenotype by TGF-beta: “N1” versus “N2” TAN. Cancer Cell. 2009;16:183–94. 19732719 10.1016/j.ccr.2009.06.017 PMC2754404

[72] Fossati G Ricevuti G Edwards SW Walker C Dalton A Rossi ML. Neutrophil infiltration into human gliomas. Acta Neuropathol. 1999;98:349–54. 10502039 10.1007/s004010051093

[73] Liang J Piao Y Holmes L. Neutrophils promote the malignant glioma phenotype through S100A4. Clin Cancer Res. 2014;20:187–98. 24240114 10.1158/1078-0432.CCR-13-1279 PMC4422653

[74] Rahbar A Cederarv M Wolmer-Solberg N. Enhanced neutrophil activity is associated with shorter time to tumor progression in glioblastoma patients. Oncoimmunology. 2016;5:e1075693. 27057448 10.1080/2162402X.2015.1075693 PMC4801436

[75] Maas RR Soukup K Fournier N. The local microenvironment drives activation of neutrophils in human brain tumors. Cell. 2023;186:4546–66.e27. 37769657 10.1016/j.cell.2023.08.043

[76] Rubenich DS de Souza PO Omizzollo N. Tumor-neutrophil crosstalk promotes in vitro and in vivo glioblastoma progression. Front Immunol. 2023;14:1183465. 37292196 10.3389/fimmu.2023.1183465 PMC10244780

[77] Yee PP Wei Y Kim SY. Neutrophil-induced ferroptosis promotes tumor necrosis in glioblastoma progression. Nat Commun. 2020;11:5424. 33110073 10.1038/s41467-020-19193-y PMC7591536

[78] Karpel-Massler G Kast RE Siegelin MD. Anti-glioma activity of dapsone and its enhancement by synthetic chemical modification. Neurochem Res. 2017;42:3382–9. 28852934 10.1007/s11064-017-2378-6

[79] Chen H Ji J Zhang L. Inflammatory responsive neutrophil-like membrane-based drug delivery system for post-surgical glioblastoma therapy. J Control Release. 2023;362:479–88. 37579976 10.1016/j.jconrel.2023.08.020

[80] Chang Y Cai X Syahirah R. CAR-neutrophil mediated delivery of tumor-microenvironment responsive nanodrugs for glioblastoma chemo-immunotherapy. Nat Commun. 2023;14:2266. 37080958 10.1038/s41467-023-37872-4 PMC10119091

[81] Chen Z Soni N Pinero G. Monocyte depletion enhances neutrophil influx and proneural to mesenchymal transition in glioblastoma. Nat Commun. 2023;14:1839. 37012245 10.1038/s41467-023-37361-8 PMC10070461

[82] Iglesias-Escudero M Arias-González N Martínez-Cáceres E. Regulatory cells and the effect of cancer immunotherapy. Mol Cancer. 2023;22:26. 36739406 10.1186/s12943-023-01714-0 PMC9898962

[83] Heimberger AB Abou-Ghazal M Reina-Ortiz C. Incidence and prognostic impact of FoxP3+ regulatory T cells in human gliomas. Clin Cancer Res. 2008;14:5166–72. 18698034 10.1158/1078-0432.CCR-08-0320

[84] Crane CA Ahn BJ Han SJ Parsa AT. Soluble factors secreted by glioblastoma cell lines facilitate recruitment, survival, and expansion of regulatory T cells: implications for immunotherapy. Neuro-Oncology. 2012;14:584–95. 22406925 10.1093/neuonc/nos014 PMC3337302

[85] Fu W Wang W Li H. Single-cell atlas reveals complexity of the immunosuppressive microenvironment of initial and recurrent glioblastoma. Front Immunol. 2020;11:835. 32457755 10.3389/fimmu.2020.00835 PMC7221162

[86] Wiencke JK Accomando WP Zheng S. Epigenetic biomarkers of T-cells in human glioma. Epigenetics. 2012;7:1391–402. 23108258 10.4161/epi.22675 PMC3528694

[87] Fong B Jin R Wang X. Monitoring of regulatory T cell frequencies and expression of CTLA-4 on T cells, before and after DC vaccination, can predict survival in GBM patients. PLoS One. 2012;7:e32614. 22485134 10.1371/journal.pone.0032614 PMC3317661

[88] Jordan JT Sun W Hussain SF DeAngulo G Prabhu SS Heimberger AB. Preferential migration of regulatory T cells mediated by glioma-secreted chemokines can be blocked with chemotherapy. Cancer Immunol Immunother. 2008;57:123–31. 17522861 10.1007/s00262-007-0336-x PMC11030978

[89] Jacobs JF Idema AJ Bol KF. Prognostic significance and mechanism of Treg infiltration in human brain tumors. J Neuroimmunol. 2010;225:195–9. 20537408 10.1016/j.jneuroim.2010.05.020

[90] Chang AL Miska J Wainwright DA. CCL2 produced by the glioma microenvironment is essential for the recruitment of regulatory t cells and myeloid-derived suppressor cells. Cancer Res. 2016;76:5671–82. 27530322 10.1158/0008-5472.CAN-16-0144 PMC5050119

[91] Li C Jiang P Wei S Xu X Wang J. Regulatory T cells in tumor microenvironment: new mechanisms, potential therapeutic strategies and future prospects. Mol Cancer. 2020;19:116. 32680511 10.1186/s12943-020-01234-1 PMC7367382

[92] Ooi YC Tran P Ung N. The role of regulatory T-cells in glioma immunology. Clin Neurol Neurosurg. 2014;119:125–32. 24582432 10.1016/j.clineuro.2013.12.004

[93] van Hooren L Handgraaf SM Kloosterman DJ. CD103+ regulatory T cells underlie resistance to radio-immunotherapy and impair CD8+ T cell activation in glioblastoma. Nat Cancer. 2023;4:665–81. 37081259 10.1038/s43018-023-00547-6 PMC10212765

[94] Long Y Tao H Karachi A. Dysregulation of glutamate transport enhances treg function that promotes vegf blockade resistance in glioblastoma. Cancer Res. 2020;80:499–509. 31723000 10.1158/0008-5472.CAN-19-1577

[95] Vlahovic G Archer GE Reap E. Phase I trial of combination of antitumor immunotherapy targeted against cytomegalovirus (CMV) plus regulatory T-cell inhibition in patients with newly-diagnosed glioblastoma multiforme (GBM). J Clin Oncol. 2016;34(15_suppl):e13518–e13518.

[96] Solomon I Amann M Goubier A. CD25-T(reg)-depleting antibodies preserving IL-2 signaling on effector T cells enhance effector activation and antitumor immunity. Nat Cancer. 2020;1:1153–66. 33644766 10.1038/s43018-020-00133-0 PMC7116816

[97] Harris AL. Hypoxia – a key regulatory factor in tumour growth. Nat Rev Cancer. 2002;2:38–47. 11902584 10.1038/nrc704

[98] Shi T Zhu J Zhang X Mao X. The role of hypoxia and cancer stem cells in development of glioblastoma. Cancers (Basel). 2023;15:2613. 37174078 10.3390/cancers15092613 PMC10177528

[99] Guo Q Fan Y Wang Q. Glioblastoma upregulates SUMOylation of hnRNP A2/B1 to eliminate the tumor suppressor miR-204-3p, accelerating angiogenesis under hypoxia. Cell Death Dis. 2023;14:147. 36810326 10.1038/s41419-023-05663-w PMC9944918

[100] Zhang L Cao Y Guo X. Hypoxia-induced ROS aggravate tumor progression through HIF-1α-SERPINE1 signaling in glioblastoma. J Zhejiang Univ Sci B. 2023;24:32–49. 36632749 10.1631/jzus.B2200269 PMC9837376

[101] Zhao S Li B Zhao R. Hypoxia-induced circADAMTS6 in a TDP43-dependent manner accelerates glioblastoma progression via ANXA2/ NF-κB pathway. Oncogene. 2023;42:138–53. 36396726 10.1038/s41388-022-02542-0

[102] Gouazé-Andersson V Delmas C Taurand M. FGFR1 induces glioblastoma radioresistance through the PLCγ/Hif1α pathway. Cancer Res. 2016;76:3036–44. 26896280 10.1158/0008-5472.CAN-15-2058

[103] Zhang G Tao X Ji B Gong J. Hypoxia-driven M2-polarized macrophages facilitate cancer aggressiveness and temozolomide resistance in glioblastoma. Oxid Med Cell Longevity. 2022;2022:1614336. 10.1155/2022/1614336 PMC9423979 36046687

[104] Zheng ZQ Chen JT Zheng MC. Nestin+/CD31+ cells in the hypoxic perivascular niche regulate glioblastoma chemoresistance by upregulating JAG1 and DLL4. Neuro-Oncology. 2021;23:905–19. 33249476 10.1093/neuonc/noaa265 PMC8168822

[105] Xiong Z Liu H He C Li X. Hypoxia contributes to poor prognosis in primary IDH-wt GBM by inducing tumor cells MES-like transformation trend and inhibiting immune cells activity. Front Oncol. 2021;11:782043. 34956900 10.3389/fonc.2021.782043 PMC8694101

[106] Cai X Wang J Wang J. Intercellular crosstalk of hepatic stellate cells in liver fibrosis: new insights into therapy. Pharmacol Res. 2020;155:104720. 32092405 10.1016/j.phrs.2020.104720

[107] Lathia JD Mack SC Mulkearns-Hubert EE Valentim CL Rich JN. Cancer stem cells in glioblastoma. Genes Dev. 2015;29:1203–17. 26109046 10.1101/gad.261982.115 PMC4495393

[108] Sampson JH Gunn MD Fecci PE Ashley DM. Brain immunology and immunotherapy in brain tumours. Nat Rev Cancer. 2020;20:12–25. 31806885 10.1038/s41568-019-0224-7 PMC7327710

[109] Park JH Lee HK. Current understanding of hypoxia in glioblastoma multiforme and its response to immunotherapy. Cancers (Basel). 2022;14:1176. 35267480 10.3390/cancers14051176 PMC8909860

[110] Domènech M Hernández A Plaja A Martínez-Balibrea E Balañà C. Hypoxia: the cornerstone of glioblastoma. Int J Mol Sci. 2021;22:12608. 34830491 10.3390/ijms222212608 PMC8620858

[111] Martínez-Reyes I Chandel NS. Cancer metabolism: looking forward. Nat Rev Cancer. 2021;21:669–80. 34272515 10.1038/s41568-021-00378-6

[112] Garcia JH Jain S Aghi MK. Metabolic drivers of invasion in glioblastoma. Front Cell Dev Biol. 2021;9:683276. 34277624 10.3389/fcell.2021.683276 PMC8281286

[113] Wolf A Agnihotri S Micallef J. Hexokinase 2 is a key mediator of aerobic glycolysis and promotes tumor growth in human glioblastoma multiforme. J Exp Med. 2011;208:313–26. 21242296 10.1084/jem.20101470 PMC3039857

[114] Zhang C Wang M Ji F. A novel glucose metabolism-related gene signature for overall survival prediction in patients with glioblastoma. Biomed Res Int. 2021;2021:8872977. 33553434 10.1155/2021/8872977 PMC7847336

[115] Sanzey M Abdul Rahim SA Oudin A. Comprehensive analysis of glycolytic enzymes as therapeutic targets in the treatment of glioblastoma. PLoS One. 2015;10:e0123544. 25932951 10.1371/journal.pone.0123544 PMC4416792

[116] Pearce EL Pearce EJ. Metabolic pathways in immune cell activation and quiescence. Immunity. 2013;38:633–43. 23601682 10.1016/j.immuni.2013.04.005 PMC3654249

[117] Yi M Jiao D Qin S Chu Q Wu K Li A. Synergistic effect of immune checkpoint blockade and anti-angiogenesis in cancer treatment. Mol Cancer. 2019;18:60. 30925919 10.1186/s12943-019-0974-6 PMC6441150

[118] Bagchi S Yuan R Engleman EG. Immune Checkpoint inhibitors for the treatment of cancer: clinical impact and mechanisms of response and resistance. Annu Rev Pathol. 2021;16:223–49. 33197221 10.1146/annurev-pathol-042020-042741

[119] Omuro A Brandes AA Carpentier AF. Radiotherapy combined with nivolumab or temozolomide for newly diagnosed glioblastoma with unmethylated MGMT promoter: an international randomized phase III trial. Neuro-Oncology. 2023;25:123–34. 35419607 10.1093/neuonc/noac099 PMC9825306

[120] Lim M Weller M Idbaih A. Phase III trial of chemoradiotherapy with temozolomide plus nivolumab or placebo for newly diagnosed glioblastoma with methylated MGMT promoter. Neuro-Oncology. 2022;24:1935–49. 35511454 10.1093/neuonc/noac116 PMC9629431

[121] Arrieta VA Chen AX Kane JR. ERK1/2 phosphorylation predicts survival following anti-PD-1 immunotherapy in recurrent glioblastoma. Nat Cancer. 2021;2:1372–86. 35121903 10.1038/s43018-021-00260-2 PMC8818262

[122] Roth P Valavanis A Weller M. Long-term control and partial remission after initial pseudoprogression of glioblastoma by anti-PD-1 treatment with nivolumab. Neuro-Oncology. 2017;19:454–6. 28039369 10.1093/neuonc/now265 PMC5464329

[123] Cloughesy TF Mochizuki AY Orpilla JR. Neoadjuvant anti-PD-1 immunotherapy promotes a survival benefit with intratumoral and systemic immune responses in recurrent glioblastoma. Nat Med. 2019;25:477–86. 30742122 10.1038/s41591-018-0337-7 PMC6408961

[124] Schalper KA Rodriguez-Ruiz ME Diez-Valle R. Neoadjuvant nivolumab modifies the tumor immune microenvironment in resectable glioblastoma. Nat Med. 2019;25:470–6. 30742120 10.1038/s41591-018-0339-5

[125] Majzner RG Mackall CL. Clinical lessons learned from the first leg of the CAR T cell journey. Nat Med. 2019;25:1341–55. 31501612 10.1038/s41591-019-0564-6

[126] Kringel R Lamszus K Mohme M. Chimeric antigen receptor T Cells in glioblastoma-current concepts and promising future. Cells. 2023;12:1770. 37443804 10.3390/cells12131770 PMC10340625

[127] Pant A Lim M. CAR-T Therapy in GBM: current challenges and avenues for improvement. Cancers (Basel). 2023;15:1249. 36831591 10.3390/cancers15041249 PMC9954019

[128] Brown CE Alizadeh D Starr R. Regression of glioblastoma after chimeric antigen receptor T-Cell therapy. N Engl J Med. 2016;375:2561–9. 28029927 10.1056/NEJMoa1610497 PMC5390684

[129] O’Rourke DM Nasrallah MP Desai A. A single dose of peripherally infused EGFRvIII-directed CAR T cells mediates antigen loss and induces adaptive resistance in patients with recurrent glioblastoma. Sci Transl Med. 2017;9:eaaa0984. 28724573 10.1126/scitranslmed.aaa0984 PMC5762203

[130] Brown CE Rodriguez A Palmer J. Off-the-shelf, steroid-resistant, IL13Rα2-specific CAR T cells for treatment of glioblastoma. Neuro-Oncology. 2022;24:1318–30. 35100373 10.1093/neuonc/noac024 PMC9340633

[131] Ahmed N Brawley V Hegde M. HER2-specific chimeric antigen receptor-modified virus-specific T cells for progressive glioblastoma: a phase 1 dose-escalation trial. JAMA Oncol. 2017;3:1094–101. 28426845 10.1001/jamaoncol.2017.0184 PMC5747970

[132] Tong L Jiménez-Cortegana C Tay AHM Wickström S Galluzzi L Lundqvist A. NK cells and solid tumors: therapeutic potential and persisting obstacles. Mol Cancer. 2022;21:206. 36319998 10.1186/s12943-022-01672-z PMC9623927

[133] Xiong Q Zhu J Zhang Y Deng H. CAR-NK cell therapy for glioblastoma: what to do next? Front Oncol. 2023;13:1192128. 37404752 10.3389/fonc.2023.1192128 PMC10315652

[134] Zhang C Burger MC Jennewein L. ErbB2/HER2-specific NK cells for targeted therapy of glioblastoma. J Natl Cancer Inst. 2016;108:djv375. 10.1093/jnci/djv375 26640245

[135] Han J Chu J Keung Chan W. CAR-engineered NK cells targeting wild-type EGFR and EGFRvIII enhance killing of glioblastoma and patient-derived glioblastoma stem cells. Sci Rep. 2015;5:11483. 26155832 10.1038/srep11483 PMC4496728

[136] Liu W Zhao Y Liu Z. Therapeutic effects against high-grade glioblastoma mediated by engineered induced neural stem cells combined with GD2-specific CAR-NK. Cell Oncol (Dordr). 2023;46:1747–62. 37420122 10.1007/s13402-023-00842-5 PMC12974676

[137] Wang J Toregrosa-Allen S Elzey BD. Multispecific targeting of glioblastoma with tumor microenvironment-responsive multifunctional engineered NK cells. Proc Natl Acad Sci USA. 2021;118:e2107507118. 34740973 10.1073/pnas.2107507118 PMC8609337

[138] Sarkaria JN Hu LS Parney IF. Is the blood–brain barrier really disrupted in all glioblastomas? A critical assessment of existing clinical data. Neuro-Oncology. 2018;20:184–91. 29016900 10.1093/neuonc/nox175 PMC5777482

[139] Song YH De R Lee KT. Emerging strategies to fabricate polymeric nanocarriers for enhanced drug delivery across blood–brain barrier: an overview. Adv Colloid Interface Sci. 2023;320:103008. 37776736 10.1016/j.cis.2023.103008

[140] Gregory JV Kadiyala P Doherty R. Systemic brain tumor delivery of synthetic protein nanoparticles for glioblastoma therapy. Nat Commun. 2020;11:5687. 33173024 10.1038/s41467-020-19225-7 PMC7655867

[141] Coluccia D Figueiredo CA Wu MY. Enhancing glioblastoma treatment using cisplatin-gold-nanoparticle conjugates and targeted delivery with magnetic resonance-guided focused ultrasound. Nanomed Nanotechnol Biol Med. 2018;14:1137–48. 10.1016/j.nano.2018.01.021 29471172

[142] Madani F Esnaashari SS Webster TJ Khosravani M Adabi M. Polymeric nanoparticles for drug delivery in glioblastoma: state of the art and future perspectives. J Control Release. 2022;349:649–61. 35878729 10.1016/j.jconrel.2022.07.023

[143] Iturrioz-Rodríguez N Sampron N Matheu A. Current advances in temozolomide encapsulation for the enhancement of glioblastoma treatment. Theranostics. 2023;13:2734–56. 37284445 10.7150/thno.82005 PMC10240814

[144] Liu H Qiu W Sun T. Therapeutic strategies of glioblastoma (GBM): The current advances in the molecular targets and bioactive small molecule compounds. Acta Pharm Sin B. 2022;12:1781–804. 35847506 10.1016/j.apsb.2021.12.019 PMC9279645

[145] Huang W Hao Z Mao F Guo D. Small molecule inhibitors in adult high-grade glioma: from the past to the future. Front Oncol. 2022;12:911876. 35785151 10.3389/fonc.2022.911876 PMC9247310

[146] Nehama D Woodell AS Maingi SM Hingtgen SD Dotti G. Cell-based therapies for glioblastoma: promising tools against tumor heterogeneity. Neuro-Oncology. 2023;25:1551–62. 37179459 10.1093/neuonc/noad092 PMC10484163

[147] Suryaprakash S Lao YH Cho HY. Engineered mesenchymal stem cell/nanomedicine spheroid as an active drug delivery platform for combinational glioblastoma therapy. Nano Lett. 2019;19:1701–5. 30773888 10.1021/acs.nanolett.8b04697

[148] Jia X Wang L Feng X. Cell membrane-coated oncolytic adenovirus for targeted treatment of glioblastoma. Nano Lett. 2023;23:11120–8. 38032110 10.1021/acs.nanolett.3c03516

[149] Pullan JE Confeld MI Osborn JK Kim J Sarkar K Mallik S. Exosomes as drug carriers for cancer therapy. Mol Pharm. 2019;16:1789–98. 30951627 10.1021/acs.molpharmaceut.9b00104

[150] Calinescu AA Kauss MC Sultan Z Al-Holou WN O’Shea SK. Stem cells for the treatment of glioblastoma: a 20-year perspective. CNS Oncol. 2021;10:CNS73. 34006134 10.2217/cns-2020-0026 PMC8162173

[151] Kim R Lee S Lee J. Exosomes derived from microRNA-584 transfected mesenchymal stem cells: novel alternative therapeutic vehicles for cancer therapy. BMB Rep. 2018;51:406–11. 29966581 10.5483/BMBRep.2018.51.8.105 PMC6130835

[152] Ghasempour E Hesami S Movahed E Keshel SH Doroudian M. Mesenchymal stem cell-derived exosomes as a new therapeutic strategy in the brain tumors. Stem Cell Res Ther. 2022;13:527. 36536420 10.1186/s13287-022-03212-4 PMC9764546

[153] Sun Y Liu G Zhang K Cao Q Liu T Li J. Mesenchymal stem cells-derived exosomes for drug delivery. Stem Cell Res Ther. 2021;12:561. 34717769 10.1186/s13287-021-02629-7 PMC8557580

[154] Rehman FU Liu Y Yang Q. Heme Oxygenase-1 targeting exosomes for temozolomide resistant glioblastoma synergistic therapy. J Control Release. 2022;345:696–708. 35341901 10.1016/j.jconrel.2022.03.036

[155] Wang K Kumar US Sadeghipour N Massoud TF Paulmurugan R. A microfluidics-based scalable approach to generate extracellular vesicles with enhanced therapeutic microrna loading for intranasal delivery to mouse glioblastomas. ACS Nano. 2021;15:18327–46. 34723509 10.1021/acsnano.1c07587

